# CT-based radiomics features for the differential diagnosis of nodular goiter and papillary thyroid carcinoma: an analysis employing propensity score matching

**DOI:** 10.3389/fonc.2024.1465941

**Published:** 2024-12-12

**Authors:** Haiming Zhang, Zhenyu Li, Fengtao Zhang, Hengguo Li

**Affiliations:** ^1^ Medical Imaging Center, The first Affiliated Hospital of Jinan University, Guangzhou, China; ^2^ Invasive Technology Department, Huazhong University of Science and Technology Union Shenzhen Hospital, Shenzhen, China

**Keywords:** nodular goiter, papillary thyroid carcinoma, computed tomography, radiomics, propensity score matching

## Abstract

**Purpose:**

This study aims to evaluate the effectiveness of CT-based radiomics features in discriminating between nodular goiter (NG) and papillary thyroid carcinoma (PTC).

**Methods:**

A retrospective cohort comprising 228 patients with nodular goiter (NG) and 227 patients with papillary thyroid carcinoma (PTC) diagnosed between January 2018 and December 2022 was consecutively enrolled. Propensity score matching (PSM) was applied to align patients with NG and PTC. A total of 851 radiomics features were extracted from CT images acquired during the arterial phase for each individual. Feature selection was carried out utilizing the least absolute shrinkage and selection operator (LASSO) logistic regression algorithm to generate the radiomics score (Rad-score). Subsequently, the Rad-score was incorporated into a multivariate logistic regression analysis to construct a radiomics nomogram for visual representation.

**Results:**

Following PSM implementation, 101 patients diagnosed with NG were matched with an equivalent number of patients diagnosed with PTC. The developed radiomics score exhibited excellent predictive performance in distinguishing between NG and PTC, with high values of AUC, sensitivity, and specificity in both the training cohort (AUC = 0.823, accuracy = 0.759, sensitivity = 0.794, specificity = 0.740) and validation cohort (AUC = 0.904, accuracy = 0.820, sensitivity = 0.758, specificity = 0.964).

**Conclusion:**

The utilization of CT-based radiomics analysis following PMS offers a quantitative and data-driven approach to enhance the accuracy of distinguishing between nodular goiter (NG) and papillary thyroid carcinoma (PTC).

## Introduction

Thyroid nodules are a prevalent clinical condition, comprising nodular goiter (NG) and papillary thyroid carcinoma (PTC) as the predominant benign and malignant subtypes, respectively (1). Traditional imaging modalities, including ultrasound and computed tomography (CT), have conventionally been employed for thyroid nodule characterization (2, 3). However, their subjective interpretation and limited quantitative assessment may contribute to diagnostic uncertainties and potential misclassifications. The overlapping clinical and imaging features of these conditions pose diagnostic challenges. Accurate differentiation between benign NG and malignant PTC is essential for guiding optimal treatment decisions and improving patient outcomes. While fine-needle aspiration (FNA) serves as the gold standard for preoperative qualitative diagnosis of thyroid nodules under ultrasound guidance, it is associated with potential sampling bias and challenges in interpreting non-specific results (4, 5).

In recent years, the emerging field of radiomics has recently become increasingly recognized as a promising tool in medical imaging analysis, offering a quantitative and data-driven approach for extracting comprehensive information from medical images (6–8). By capturing subtle imaging features and quantifying intricate patterns within the tumor microenvironment, radiomics holds the potential to provide deeper insights into the underlying tissue characteristics and improve the diagnostic accuracy of thyroid nodules (9–11). The utilization of radiomics in thyroid imaging represents a paradigm shift towards precision medicine, enabling a more refined and personalized approach to the diagnosis and management of thyroid diseases (12, 13).

While previous studies (13, 14) have showcased the effectiveness of radiomics in oncology for tumor characterization, prognostication, and treatment response assessment, the application of CT-based radiomics in distinguishing between nodular goiter (NG) and papillary thyroid carcinoma (PTC) remains relatively limited. This study aims to bridge this gap by investigating the role of CT-based radiomics in distinguishing between NG and PTC through the development of a radiomics nomogram based on advanced imaging analysis techniques. The CT arterial phase images demonstrate the most pronounced contrast enhancement disparities between benign and malignant thyroid nodules, which are essential for precise radiomics analysis. By leveraging the wealth of quantitative information extracted from CT images of arterial phase, we seek to enhance the accuracy, objectivity, and predictive capability of thyroid nodule differentiation, ultimately contributing to improved patient care and clinical decision-making in thyroid pathology.

Propensity score matching (PSM) is a commonly used statistical method in observational studies to eliminate confounding bias when randomization is not feasible (15). As an alternative to multiple regression analysis, PSM matches treated subjects with control subjects who exhibit similar treatment propensities based on preexisting covariates that influence treatment selection, thereby reducing the impact of confounders. This study employed PSM to evaluate the feasibility of CT-based radiomic features in differentiating nodular goiter (NG) from papillary thyroid carcinoma (PTC).

## Materials and methods

### Study population

The retrospective cross-sectional study received approval from the Ethics Committee of the First Affiliated Hospital of Jinan University, with a waiver of the requirement for informed consent from participants. The study enrolled a cohort comprising 228 patients diagnosed with nodular goiter and 227 patients with papillary thyroid carcinoma, as confirmed by postoperative pathology. Inclusion criteria comprised: (i) preoperative contrast-enhanced CT imaging of the neck; and (ii) histological confirmation of thyroid nodules as nodular goiter or papillary thyroid carcinoma. Exclusion criteria included: (i) incomplete clinical laboratory data; (ii) concurrent presence of benign and malignant thyroid nodules; (iii) history of prior surgical interventions or medication treatments before CT scans; and (iv) presence of CT imaging artifacts (e.g., nodules with coarse calcifications) or thyroid nodules with a maximum diameter of less than 3 mm, which may hinder accurate delineation of the region of interest. Following the rigorous application of the aforementioned inclusion and exclusion criteria, a cohort of 160 patients diagnosed with nodular goiter and 173 patients with papillary thyroid cancer were meticulously selected for analysis.

**Figure 1:**
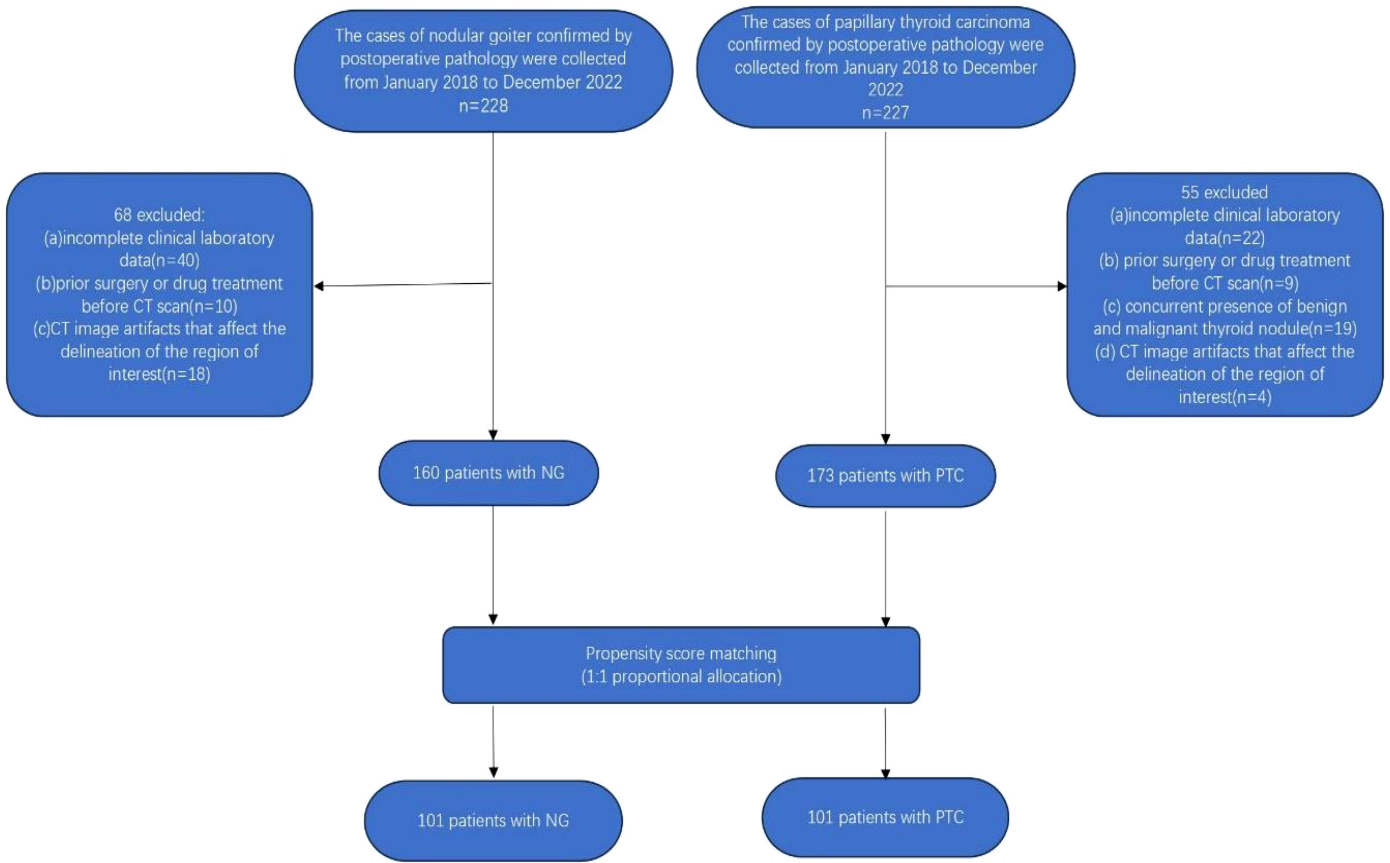
Flow diagram showing the selection of the study population.

Subsequently, a meticulous 1:1 propensity score matching analysis was employed to harmonize the baseline clinical data of patients with nodular goiter and papillary thyroid carcinoma. This meticulous process culminated in the inclusion of a carefully matched cohort comprising 101 patients with nodular goiter (16 males, 85 females; mean age 41.515 ± 14.775 years; age range: 14-79 years) and 101 patients with papillary thyroid carcinoma (11 males, 90 females; mean age 41.139 ± 11.843 years; age range: 17-71 years). After propensity score matching analysis (PSM), the remaining 202 patients were randomly allocated into a training cohort(n=141) and a validation cohort(n=61) in a ratio of 7:3. The detailed patient selection process is visually depicted in **Figure 1**.

### Radiomics workflow

**Figure 2:**
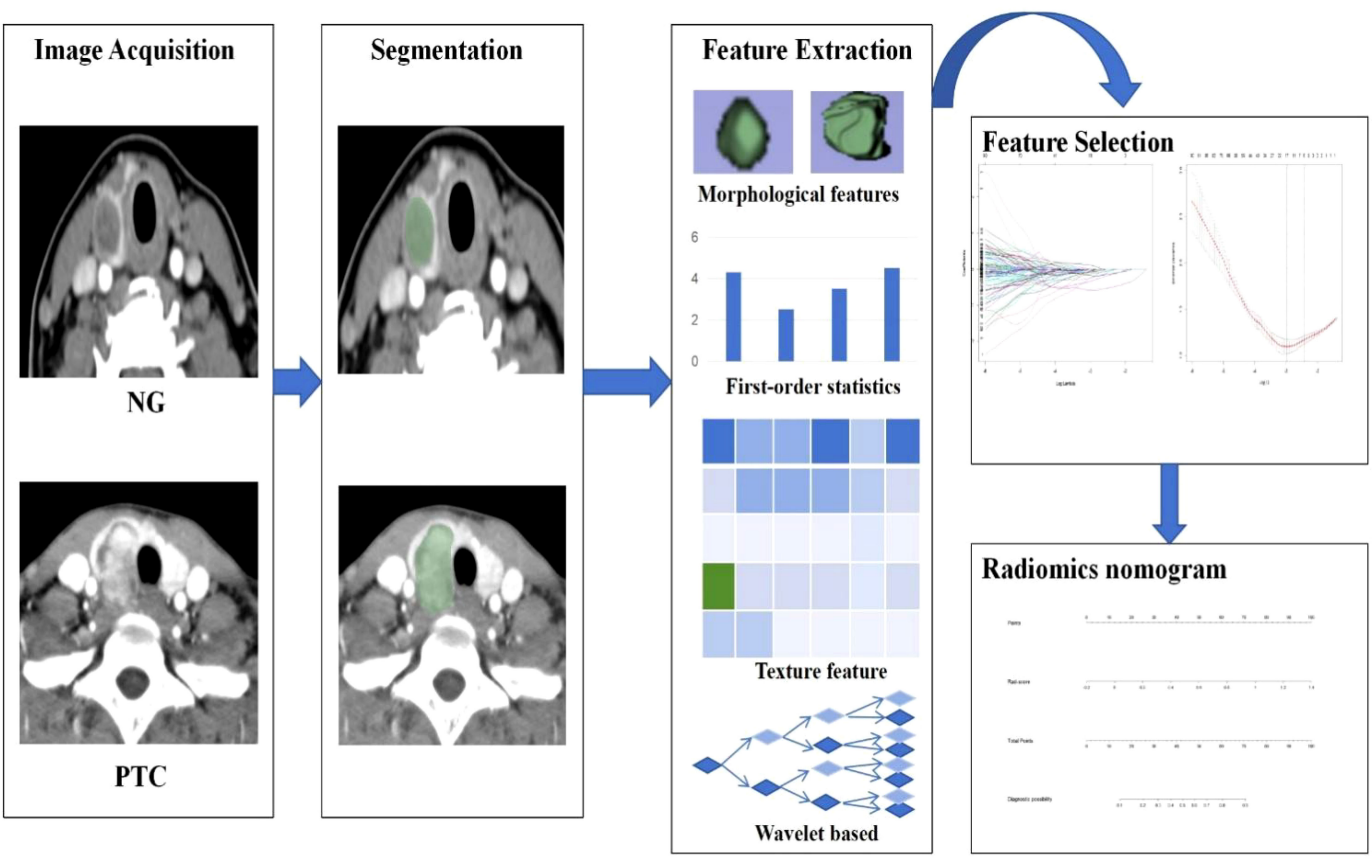
The workflow of CT-based radiomics includes: Image acquisition; Segmentation; Feature extraction; Feature selection; Development of a Radiomics Nomogram.

The radiomics workflow entails the sequential stages of CT image acquisition, segmentation, feature extraction and selection, and the development of radiomics nomogram. The detailed workflow of Radiomics is illustrated in **Figure 2**.

### CT image acquisition

We performed enhanced CT imaging of the neck utilizing a Toshiba 16-row or 320-row 640-slice spiral CT system manufactured. Standard clinical scanning protocols were adhered to, with parameters configured at 120 KV, 200-350 mA, a pitch of 0.8, and a slice thickness of 3mm by established clinical practice guidelines. Patients were positioned in the supine orientation, and the scanning coverage extended from the oropharynx to the superior margin of the clavicle. Iodixanol (350mg iodine/ml) is utilized as a contrast agent, with the standard adult dosage being 50 ml and the pediatric dosage being 1 ml/kg. An automatic injector is employed, with the injection rate set at 2-4 ml/sec. The contrast injection is administered using an aorta tracking method, which continuously monitors the concentration of the contrast agent in the aorta and automatically initiates scanning once a preset threshold is reached. This approach enhances the quality of arterial phase images and reduces individual variations. However, the arterial phase of CT imaging is prone to artifacts, particularly high-density artifacts from the brachiocephalic vein. These can be mitigated by adjusting the rate of contrast agent injection, optimizing scan timing, or employing iterative reconstruction techniques. Subsequently, all CT images were extracted from the Picture Archiving and Communication System (PACS) in the “DICOM” format for analysis and interpretation in this study.

### Segmentation, feature extraction, and selection

The initial step involves segmenting thyroid nodules. The CT images in “DICOM” format were meticulously imported into the 3D-Slicer software (version 4.10.2, open-source; https://www.slicer.org/) by two experienced radiologists for precise delineation of thyroid nodules. The region of interest (ROI) was carefully outlined layer by layer to ensure utmost precision, with meticulous attention to tumor morphology and boundaries. Automated generation of a three-dimensional volumetric image was facilitated through the use of level tracing and slice interpolation functions, resulting in the establishment of a volume of interest (VOI).

The subsequent step entails feature extraction and selection, leveraging Radiomics, an embedded plug-in within the 3D-Slicer software, to extract radiomics features. These features encompass first-order characteristics, morphological attributes, GLCM (Gray Level Co-occurrence Matrix), GLDM (Gray Level Dependence Matrix), GRLM (Gray Level Run Length Matrix), GLSZM (Gray Level Size Zone Matrix), NGTDM (Neighborhood Gray Tone Difference Matrix), and Wavelet small wave features.

To assess the inter-observer reliability among different observers and machines, two radiologists, blinded to the pathological results, were randomly selected to independently segment ROIs in 40 cases. For intra-observer reliability, the first reader re-extracted features after a one-month interval. The intraclass correlation coefficient (ICC) was used to evaluate the consistency and reproducibility of the extracted radiomics features. Radiomics features with an ICC greater than 0.75 were retained for subsequent studies.

Prior to dimensionality reduction and feature selection in radiomics analysis, data standardization was performed through Z-score normalization. The redundant features, which had a Spearman correlation coefficient exceeding 0.9, were excluded. The open-source R software was used concurrently for radiomics feature selection. Statistically significant features distinguishing between the two groups were identified through the application of the Mann-Whitney U test, Spearman correlation analysis, and Minimum Redundancy Maximum Relevance (mRMR). The redundant features, which had a Spearman correlation coefficient exceeding 0.9, were excluded.

### Development of CT radiomics signature and nomogram

Meanwhile, the Lasso algorithm was employed to select the most informative features for distinguishing between NG and PTC in the training set. To mitigate overfitting, a fivefold cross-validation was performed iteratively 100 times. The resulting radiomics score (rad-score) was computed by multiplying the corresponding coefficients. Subsequently, a radiomics nomogram was constructed using multivariate logistic regression analysis within the training cohort.

### Statistical analysis

The statistical analysis was performed using SPSS version 26.0 and R version 4.3.0 for data analysis. The clinical baseline data of patients with nodular goiter and papillary thyroid carcinoma were matched in a 1:1 ratio through propensity score matching analysis, with a caliper value set at 0.05. Quantitative data that followed a normal distribution were presented as mean ± standard deviation (SD), while non-normally distributed quantitative data were described using the median and interquartile range (IQR). To compare groups, we utilized the independent sample t-test for normally distributed data and the Mann-Whitney U test for non-normally distributed data. Categorical data were reported in terms of frequency and percentage. In SPSS version 26.0, univariate analysis was conducted using the chi-square test for categorical variables. Lasso regression analysis and multivariate logistic regression analysis were performed in R utilizing the “glmnet” and “caret” packages. The model’s predictive performance and clinical utility were evaluated by generating ROC curves, calibration curves, and decision curves. Statistical significance was considered when *P* < 0.05.

## Results

### Clinical characteristics

**Figure 3:**
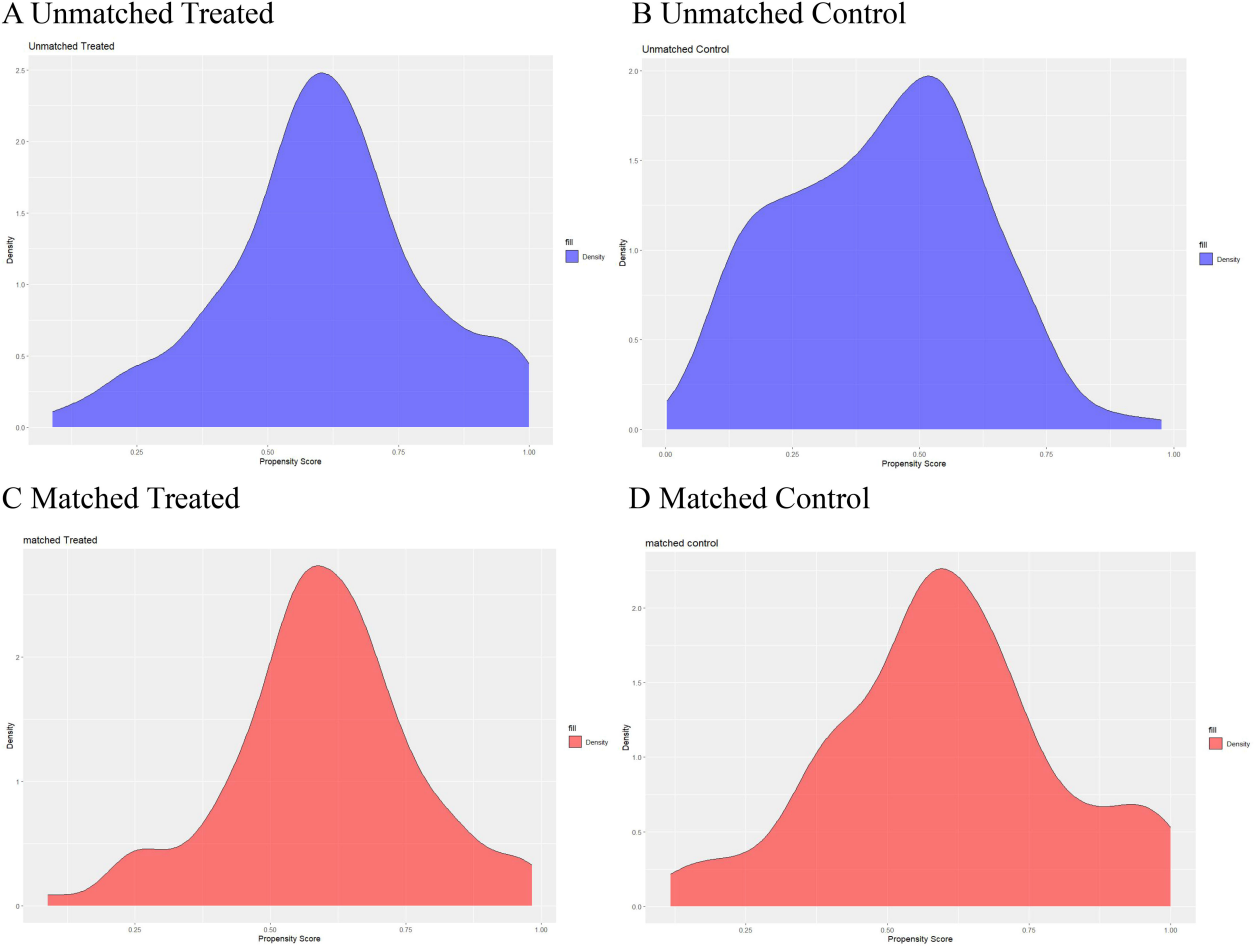
Propensity scores of the baseline characteristics before and after matching. Distribution of propensity scores before **(A, B)** and after matching **(C, D)**.

The clinical characteristics of patients with nodular goiter (NG) and papillary thyroid carcinoma (PTC) before and after propensity score matching (PSM) are detailed in **Table 1** and the propensity score distributions are shown in **Figure 3**. A total of 160 cases of nodular goiter and 173 cases of papillary thyroid carcinoma were included in the study. Baseline clinical variables were harmonized using PSM to mitigate selection bias. Subsequent to PSM, 101 NG patients were matched with 101 PTC patients based on their clinical profiles. Prior to matching, significant differences in gender, FT4, TSH, ANTI-TG, and TG were observed between the NG and PTC groups (*p* < 0.05). However, following matching, no statistically significant clinical disparities were detected between the NG and PTC cohorts (*p* > 0.05).

**Table 1:** Clinical characteristics of patients with NG and PTC before and after propensity score matching.

Characteristics	Before match			After match		
	NG (n=160)	PTC(n=173)	P-value	NG (n=101)	PTC(n=101)	P-value
**Age, years (mean ± SD)**	43.2 ± 14.0	39.2 ± 11.8	0.007	41.5 ± 14.8	41.1 ± 11.8	0.842
**Gender**			0.039			0.409
**Male**	20	35		16	11	
**Female**	140	138		85	90	
**FT3, pmol/L(mean ± SD)**	5.19 ± 0.77	5.32 ± 0.86	0.122	5.27 ± 0.71	5.22 ± 0.76	0.637
**FT4, pmol/L(mean ± SD)**	13.14 ± 13.14	11.61 ± 11.61	<0.001	12.03 ± 2.32	12.03 ± 2.16	0.651
**TSH, mIU/L(M(P25, P75))**	1.17(0.75, 1.76)	1.38(1.022,1.97)	0.004	1.25(0.78,1.86)	1.40(1.00,1.92)	0.181
**ANTI-TG, IU/mL(M(P25, P75))**	0.60(0.09,15.00)	0.21(0.00,6.81)	0.003	0.45(0.07,3.75)	0.20(0.00,3.04)	0.061
**ANTI-TPO, IU/mL(M(P25, P75))**	2.93(0.62, 28.00)	1.71(0.57,21.55)	0.384	1.69(0.45,28.00)	1.45(0.52,13.37)	0.824
**TG, ng/mL(mean ± SD)**	76.89 ± 105.83	41.03 ± 93.76	<0.001	49.02 ± 79.39	53.45 ± 114.73	0.750

FT3, Free Triiodothyronine; FT4, Free Thyroxine; TSH, Thyroid Stimulating Hormone; ANTI-TG, Anti-Thyroglobulin Antibody; ANTI-TPO, Anti-Thyroid Peroxidase Antibody; TG, Thyroglobulin.

### Construction of radiomics signature

**Figure 4:**
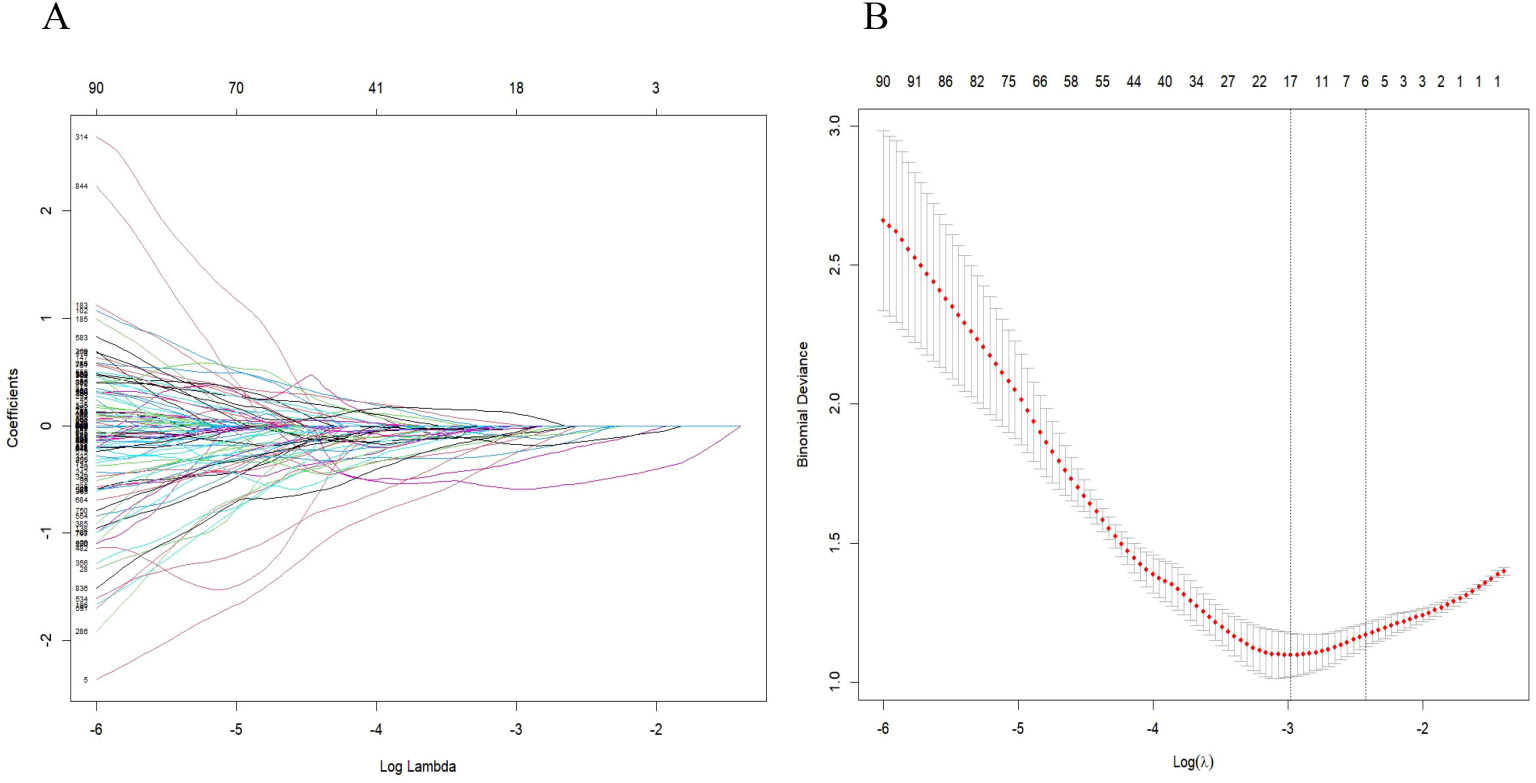
Radiomic feature selection was conducted using the parametric method known as the least absolute shrinkage and selection operator (LASSO). **(A)** Tuning parameter (λ) selection in the LASSO model was performed through 5-fold cross-validation based on minimum criteria. **(B)** The LASSO coefficient profiles of the radiomics features were analyzed, highlighting six resulting features with nonzero coefficients in the plot.

A comprehensive array of 851 radiomics features was extracted from CT images of arterial phase-enhanced. The six most valuable features were identified through Lasso logistic regression (**Figure 4**). These selected features were then linearly combined with their respective coefficients to compute the radiomics scores(rad-score).

Rad-score=−0.01243199−0.04367516*wavelet-HLL_firstorder-Uniformity

−0.50841517*wavelet-HLL_glrlm-GrayLevelNonUniformityNormalized

−0.05779006*wavelet-LHL_firstorder_Uniformity

−0.16264499*wavelet-LHH_glrlm_LongRunLowGrayLevelEmphasis

−0.13338604*wavelet-LLH_glrlm_GrayLevelNonUniformityNormalized

−0.02678135*wavelet-LLH_glszm_SizeZoneNonUniformity

The differences in the rad-score values between NG and PTC were statistically significant in both the training and validation cohorts (p<0.001, **Table 2**).

**Table 2:** Rad-score of patients in the NG and PTC groups in the training and validation cohorts.

	Training cohort		P-value	Validation cohort		P-value
	NG (n=68)	PTC(n=73)		NG (n=33)	PTC (n=28)	
Rad-score (mean ± SD)	0.340 ± 0.266	0.663 ± 0.239	<0.001	0.295 ± 0.203	0.707 ± 0.213	<0.001

### Development and performance of radiomics nomogram

**Figure 5:**
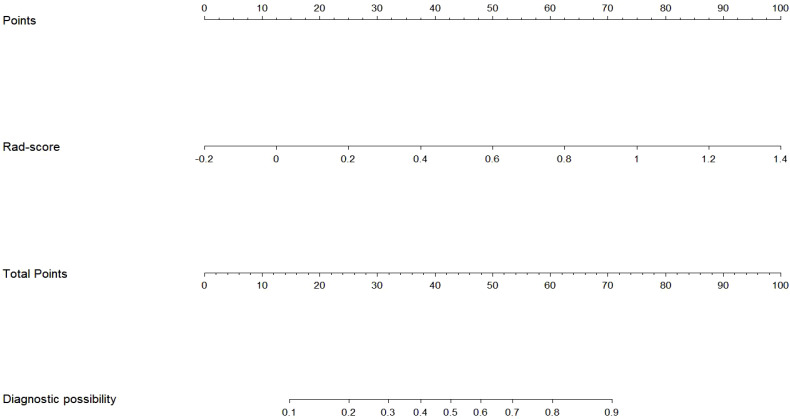
The radiomics nomogram was developed based on the radiomics score in the training cohort.

The Rad-score served as an independent predictor for distinguishing between nodular goiter (NG) and papillary thyroid carcinoma (PTC) through multivariate logistic regression. Subsequently, a radiomics nomogram was developed to personalize discrimination (**Figure 5**). Lower risk values on the nomogram indicate a higher likelihood of NG, while higher risk values suggest a greater propensity towards PTC.

**Figure 6:**
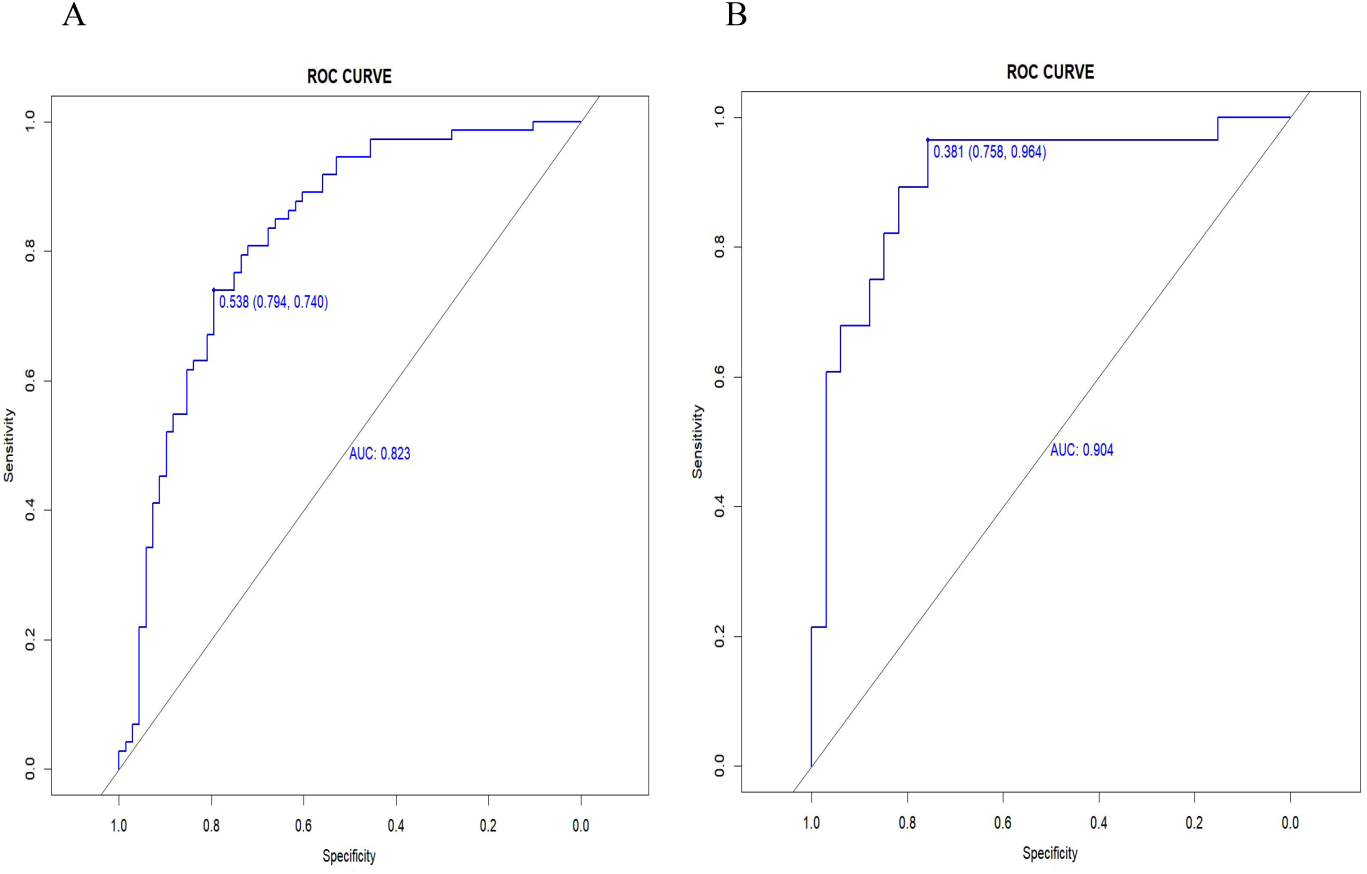
The AUC of the radiomics nomogram in the training cohort **(A)** and validation cohort **(B)**.

**Figure 7:**
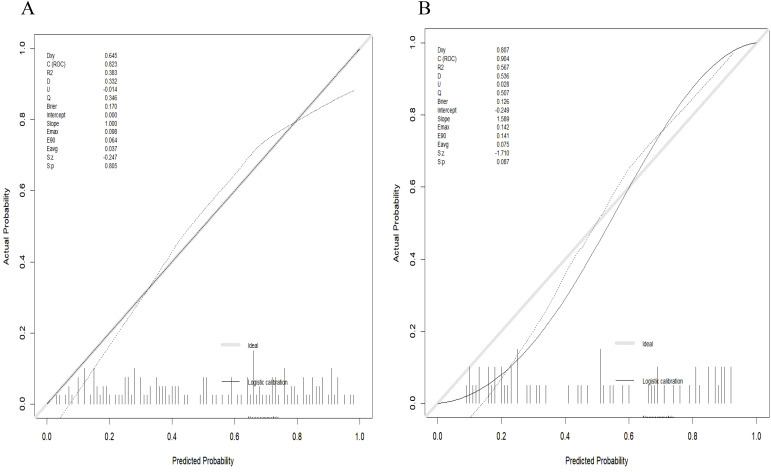
Calibration curve of the radiomics nomogram. **(A)** Calibration curve of the nomogram in the training cohort showed a nonsignificant statistic (p = 0.782) in the Hosmer-Lemeshow test; **(B)** Calibration curve of the nomogram in the validation cohort displayed a nonsignificant statistic (p = 0.710) in the Hosmer-Lemeshow test.

**Figure 8:**
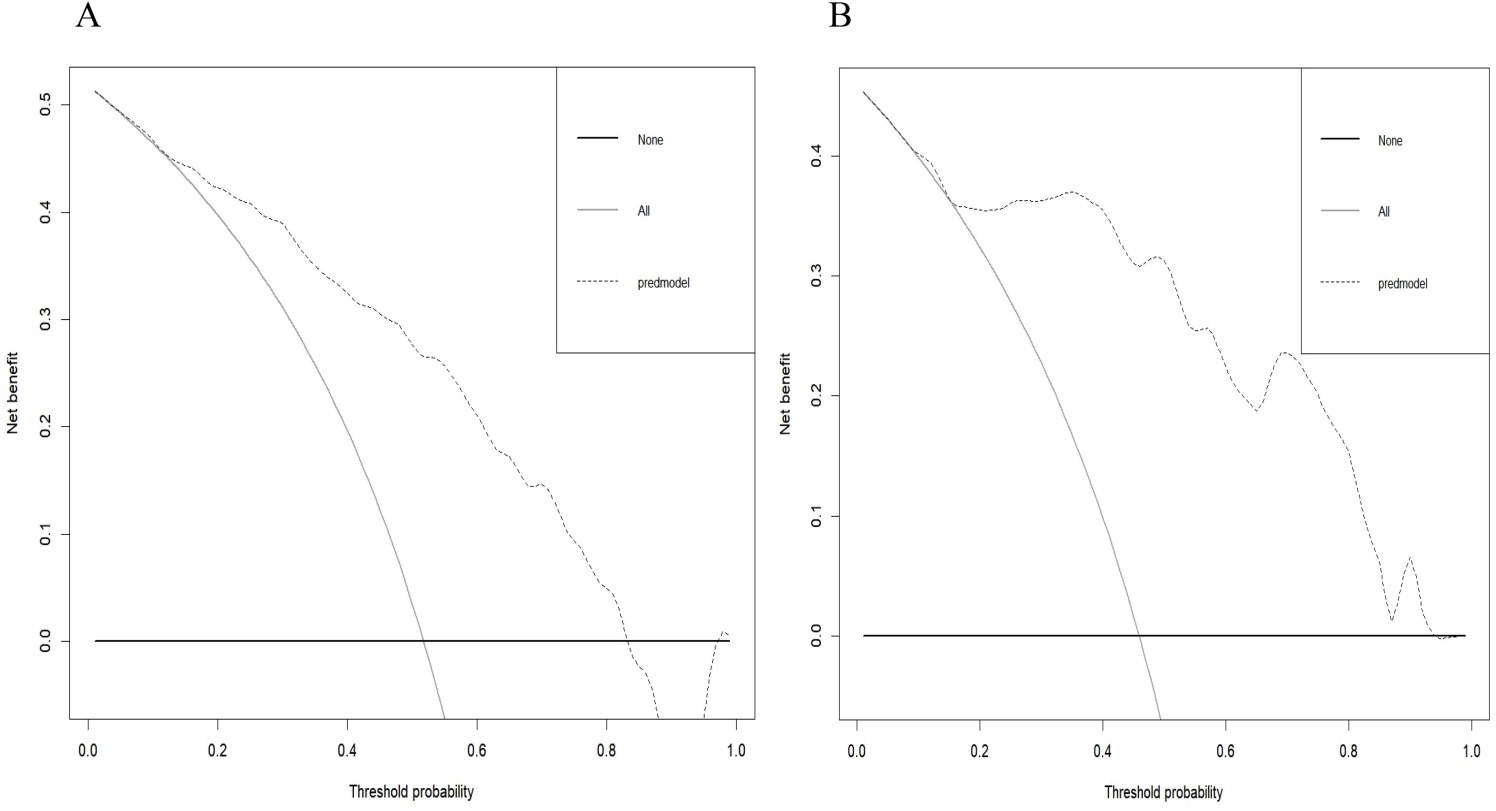
Decision curve of the radiomics nomogram in the training cohort **(A)** and validation cohort **(B)**. The decision curve demonstrated that if the threshold probability is within the range of 20% to 80%, the application of radiomics nomogram to differentiate NG from PTC adds more benefit than treating all or none of the patients.

Radiomics nomogram demonstrated excellent predictive performance in both the training cohort (AUC = 0.823, accuracy = 0.759, sensitivity = 0.794, specificity = 0.740) and the validation cohort (AUC = 0.904, accuracy = 0.820, sensitivity = 0.758, specificity = 0.964), as depicted in (**Figure 6**, **Table 3**). Calibration curves for the training and validation cohorts showed close alignment between the calibrated prediction curve and the ideal standard curve, indicating the accurate predictive ability of the Radiomics nomogram for PTC occurrence (**Figure 7**). Decision curve analysis on both the training and validation cohorts revealed substantial net benefits for patients at probability threshold values of 0.2-0.8 using the radiomics nomogram, underscoring its significant clinical applicability (**Figure 8**).

**Table 3:** Diagnostic performances of the radiomics nomogram in training and validation cohorts.

	AUC (95%CI)	Accuracy	sensitivity	specificity
Training cohort	0.823(0.752-0.893)	0.759	0.794	0.794
Validation cohort	0.904(0.822-0.985)	0.820	0.758	0.964

## Discussion

This research presents a significant progress in the field of thyroid imaging by introducing a CT-based radiomics nomogram to distinguish between nodular goiter (NG) and papillary thyroid carcinoma (PTC). Propensity score matching (PSM) is a commonly employed statistical technique in observational clinical research or clinical trial data, aimed at addressing confounding biases and achieving comparable outcomes to those of randomized controlled trials throughout the entire study design process (16). In the investigation by Li et al. (17), post Propensity Score Matching (PSM), their findings suggested that radiomics analysis of CT images demonstrates a satisfactory level of accuracy in differentiating focal autoimmune pancreatitis (fAIP) from pancreatic ductal adenocarcinoma (PDAC). This study is the first to apply Propensity Score Matching (PSM) in Thyroid radiomics research to control for clinical confounding biases. By incorporating radiomics analysis with propensity score matching (PSM) methodology, a more precise and reliable approach has been developed to differentiate these two thyroid conditions.

Currently, significant advancements have been made in US-based radiomics research for distinguishing between benign and malignant thyroid nodules and predicting neck lymph node metastasis of malignant thyroid cancers; however, the exploration of CT-based radiomics technology in thyroid imaging remains limited (18, 19). US radiomics studies typically focus on the largest slice of a single thyroid lesion, which may result in loss of critical tumor information and be susceptible to operator subjectivity. In contrast, CT-based radiomics allows for the delineation of the tumor contour layer by layer, capturing comprehensive tumor information and significantly minimizing the impact of subjective factors.

Previous studies have made some progress in differentiating nodular goiter (NG) and papillary thyroid carcinoma (PTC) using CT texture analysis and radiomics models. Guo et al. (20) utilized CT-based texture analysis and identified that by setting the entropy value at 6.55 as the threshold for distinguishing between benign and malignant thyroid nodules, the AUC was calculated to be 0.716 (95% CI: 0.585-0.847, P=0.005), with corresponding sensitivity and specificity values of 75.0% and 62.5%, respectively. Li et al. (21) demonstrated that a radiomics model, utilizing 3D features extracted from CT images of arterial phase, outperformed a panel of experienced radiologists in differentiating NG and PTC achieved impressive AUCs of 0.889 and 0.887 in the training and validation sets respectively. However, these studies are retrospective in nature, introducing selection bias, and generally demonstrate poor performance in distinguishing between the two groups using CT texture analysis alone. This study utilized propensity score matching (PSM) to mitigate the impact of clinical confounding factors, with the aim of assessing the potential application of CT Radiomics in distinguishing between nodular goiter and papillary thyroid carcinoma. The radiomics nomogram’s performance was visualized using ROC curves, and its clinical utility was further validated through calibration curves and decision curve analysis.

In order to achieve the visualization of the Rad-score, we developed a radiomics nomogram to aid clinicians in personalized differentiation between nodular goiter (NG) and papillary thyroid carcinoma (PTC). The ability of the radiomics nomogram to stratify patients based on their risk of either NG or PTC highlights its potential for individualized patient care and decision-making. The strong predictive performance demonstrated by the radiomics score, as evidenced by the high sensitivity and specificity values in both the training (sensitivity = 0.794, specificity = 0.740) and validation groups (sensitivity = 0.758, specificity = 0.964), underscores its clinical utility and reliability. Moreover, the calibration curves and decision curve analysis further validate the robustness and clinical relevance of the radiomics score. The radiomics nomogram’s ability to accurately discriminate between NG and PTC contributes to improved patient outcomes and treatment strategies.

The comprehensive evaluation of 851 radiomics features extracted from CT images of arterial phase-enhanced in this study underscores the depth and complexity of the information captured through radiomics analysis. The arterial phase captures peak contrast enhancement, facilitating the visualization of vascular structures within the thyroid. This is crucial for identifying vascular proliferation associated with malignancy in papillary thyroid carcinoma (PTC) and is particularly significant when compared to the more uniform enhancement pattern observed in nodular goiter (NG). By identifying the six most valuable radiomics features and incorporating them into the radiomics scores, we have created a powerful tool for clinicians to leverage in the diagnostic process. The radiomics scores derived from the selected features and their corresponding coefficients provide a quantitative measure that enhances the objectivity and precision of the diagnostic model.

The discovery in this study that the radiomics score is primarily composed of wavelet features signifies that image features derived from wavelet characteristics effectively encapsulate subtle textural intricacies within medical images. This underscores the capacity of wavelet-based radiomics features to accurately represent fine textural details in thyroid imaging, thereby enriching the realm of medical imaging with comprehensive and precise information crucial for disease diagnosis, prognosis evaluation, and treatment monitoring (22, 23).

While this study contributes valuable insights into the application of CT-based radiomics in the differential diagnosis of nodular goiter (NG) and papillary thyroid carcinoma (PTC), several limitations should be acknowledged. First, this study was conducted with a limited sample size, which may impact the generalizability of the findings. Future studies with larger and more diverse patient cohorts are warranted to validate the robustness of the radiomics nomogram. Second, this research was conducted in a single center, which may introduce center-specific biases and limit the external validity of the results. Multi-center studies are needed to enhance the generalizability of the radiomics model. Third, despite the use of propensity score matching to adjust for potential biases, the retrospective nature of the study may still introduce selection bias. Prospective studies with well-defined inclusion criteria can mitigate this limitation. In addition, this study focused on CT arterial phase-enhanced images for radiomics analysis. Incorporating other imaging modalities, such as MRI or ultrasound, may provide a more comprehensive evaluation of thyroid nodules.

In conclusion, the application of PSM facilitates the development and evaluation of a radiomics nomogram for distinguishing between NG and PTC, thereby advancing personalized and evidence-based healthcare in the field of thyroid imaging. The integration of advanced imaging analysis techniques with statistical methodologies has the potential to enhance diagnostic practices and improve patient outcomes in thyroid disease management. Further research and clinical validation are warranted to fully realize the clinical impact and potential of radiomics in thyroid pathology.

## Data Availability

The original contributions presented in the study are included in the article/supplementary material. Further inquiries can be directed to the corresponding author/s.

## References

[1] Baloch ZW Asa SL Barletta JA Ghossein RA Juhlin CC Jung CK. Overview of the 2022 WHO classification of thyroid neoplasms. Endocrine pathology. (2022) 33:27–63. doi: 10.1007/s12022-022-09707-3 35288841

[2] Traylor KS. Computed tomography and MR imaging of thyroid disease. Radiol Clin North Am. (2020) 58:1059–70. doi: 10.1016/j.rcl.2020.07.004 33040848

[3] Kim JY Jung SL Kim MK Kim TJ Byun JY. Differentiation of benign and Malignant thyroid nodules based on the proportion of sponge-like areas on ultrasonography: imaging-pathologic correlation. Ultrasonography. (2015) 34:304–11. doi: 10.14366/usg.15016 PMC4603205 26006056

[4] Alexander EK Cibas ES. Diagnosis of thyroid nodules. Lancet Diabetes Endocrinol. (2022) 10:533–9. doi: 10.1016/s2213-8587(22)00101-2 35752200

[5] Kezlarian B Lin O. Artificial intelligence in thyroid fine needle aspiration biopsies. Acta Cytol. (2021) 65:324–9. doi: 10.1159/000512097 PMC8491503 33326953

[6] Bera K Braman N Gupta A Velcheti V Madabhushi A. Predicting cancer outcomes with radiomics and artificial intelligence in radiology. Nat Rev Clin Oncol. (2022) 19:132–46. doi: 10.1038/s41571-021-00560-7 PMC9034765 34663898

[7] Guiot J Vaidyanathan A Deprez L Zerka F Danthine D Frix AN. A review in radiomics: Making personalized medicine a reality via routine imaging. Med Res Rev. (2022) 42:426–40. doi: 10.1002/med.21846 34309893

[8] Mayerhoefer ME Materka A Langs G Häggström I Szczypiński P Gibbs P. Introduction to radiomics. J Nucl Med. (2020) 61:488–95. doi: 10.2967/jnumed.118.222893 PMC9374044 32060219

[9] Gao X Ran X Ding W. The progress of radiomics in thyroid nodules. Front Oncol. (2023) 13:1109319. doi: 10.3389/fonc.2023.1109319 36959790 PMC10029726

[10] Zhang C Liu D Huang L Zhao Y Chen L Guo Y. Classification of thyroid nodules by using deep learning radiomics based on ultrasound dynamic video. J Ultrasound Med. (2022) 41:2993–3002. doi: 10.1002/jum.16006 35603714

[11] Guo SY Zhou P Zhang Y Jiang LQ Zhao YF. Exploring the value of radiomics features based on B-mode and contrast-enhanced ultrasound in discriminating the nature of thyroid nodules. Front Oncol. (2021) 11:738909. doi: 10.3389/fonc.2021.738909 34722288 PMC8551634

[12] Gul M Bonjoc KC Gorlin D Wong CW Salem A La V. Diagnostic utility of radiomics in thyroid and head and neck cancers. Front Oncol. (2021) 11:639326. doi: 10.3389/fonc.2021.639326 34307123 PMC8293690

[13] Lu WW Zhang D Ni XJ. A review of the role of ultrasound radiomics and its application and limitations in the investigation of thyroid disease. Med Sci Monit. (2022) 28:e937738. doi: 10.12659/msm.937738 36258648 PMC9587688

[14] Inchingolo R Maino C Cannella R Vernuccio F Cortese F Dezio M. Radiomics in colorectal cancer patients. World J Gastroenterol. (2023) 29:2888–904. doi: 10.3748/wjg.v29.i19.2888 PMC10237092 37274803

[15] Kane LT Fang T Galetta MS Goyal DKC Nicholson KJ Kepler CK. Propensity score matching: A statistical method. Clin Spine Surg. (2020) 33:120–2. doi: 10.1097/bsd.0000000000000932 31913173

[16] Wang J. To use or not to use propensity score matching? Pharm Stat. (2021) 20:15–24. doi: 10.1002/pst.2051 32776719

[17] Li J Liu F Fang X Cao K Meng Y Zhang H. CT radiomics features in differentiation of focal-type autoimmune pancreatitis from pancreatic ductal adenocarcinoma: A propensity score analysis. Acad Radiol. (2022) 29:358–66. doi: 10.1016/j.acra.2021.04.014 34108115

[18] Tong Y Li J Huang Y Zhou J Liu T Guo Y. Ultrasound-based radiomic nomogram for predicting lateral cervical lymph node metastasis in papillary thyroid carcinoma. Acad Radiol. (2021) 28:1675–84. doi: 10.1016/j.acra.2020.07.017 32782219

[19] Pang L Yang X Zhang P Ding L Yuan J Liu H. Development and validation of a nomogram based on multimodality ultrasonography images for differentiating Malignant from benign american college of radiology thyroid imaging, reporting and data system (TI-RADS) 3-5 thyroid nodules. Ultrasound Med Biol. (2024) 50:557–63. doi: 10.1016/j.ultrasmedbio.2023.12.020 38262884

[20] Guo W Bai W Liu J Luo D Yuan H. Can contrast-enhancement computed tomography texture and histogram analyses help to differentiate Malignant from benign thyroid nodules? Jpn J Radiol. (2020) 38:1135–41. doi: 10.1007/s11604-020-01018-z 32661879

[21] Li Z Zhang H Chen W Li H. Contrast-enhanced CT-based radiomics for the differentiation of nodular goiter from papillary thyroid carcinoma in thyroid nodules. Cancer Manage Res. (2022) 14:1131–40. doi: 10.2147/cmar.S353877 PMC8943619 35342307

[22] Chetan MR Gleeson FV. Radiomics in predicting treatment response in non-small-cell lung cancer: current status, challenges and future perspectives. Eur Radiol. (2021) 31:1049–58. doi: 10.1007/s00330-020-07141-9 PMC7813733 32809167

[23] Whybra P Zwanenburg A Andrearczyk V Schaer R Apte AP Ayotte A. The image biomarker standardization initiative: standardized convolutional filters for reproducible radiomics and enhanced clinical insights. Radiology. (2024) 310:e231319. doi: 10.1148/radiol.231319 38319168 PMC10902595

